# Dermoscopy of Congenital Langerhans Cell Histiocytosis

**DOI:** 10.5826/dpc.1003a63

**Published:** 2020-06-29

**Authors:** Ramon Pigem, Ariann Dyer, Sebastian Podlipnik, Cristina Carrera, Susana Puig, Juan Ferrando

**Affiliations:** 1 Department of Dermatology, Hospital Clínic, Universitat de Barcelona, Barcelona, Spain

**Keywords:** Langerhans cell, histiocytosis, solar eclipse, dermoscopy

## Introduction

Langerhans cell histiocytosis (LCH) is a dysfunction and proliferation of Langerhans cells that cause a polymorphic disease depending on the affected organs [1]. We present the dermoscopic appearance of congenital LCH.

## Case Presentation

A healthy-appearing preterm newborn presented with multiple erythematous and violaceous maculopapular and nodular skin lesions scattered over trunk, face, scalp, nails, palms, and soles. The lesions were purplish-brownish, elastic, and not ulcerated. No other abnormalities were found.

**Figure 1:**
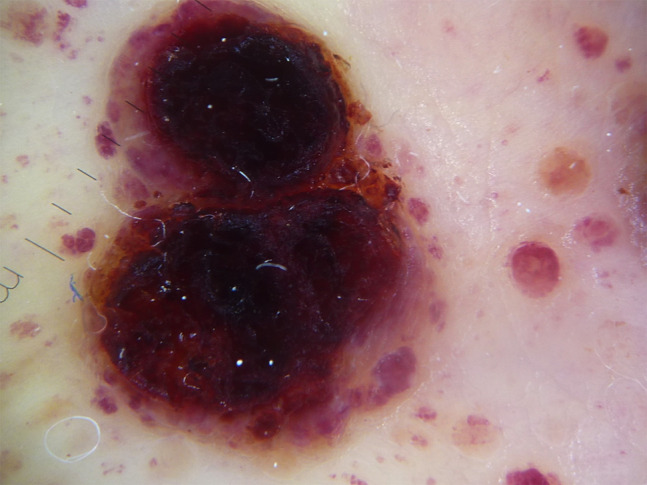
Dermoscopic examination of a lesion in the fully developed stage. The dark pigmentation in the center and the violaceous vessels on the periphery resemble a solar eclipse over a starry sky. Note the burgundy color of the vessels.

**Figure 2:**
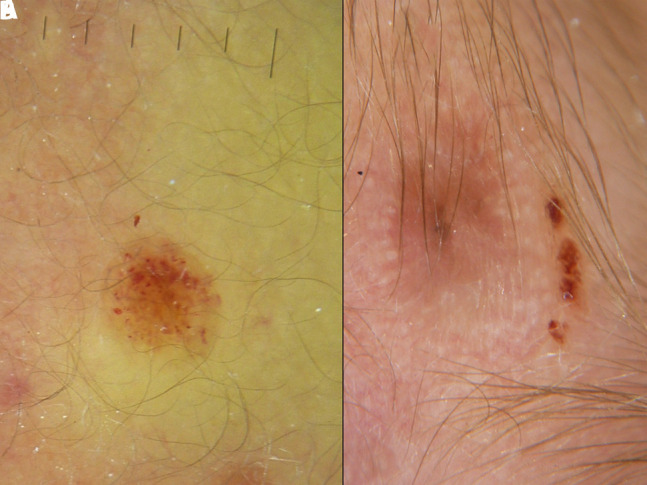
Dermoscopic examination of 2 different lesions. (A) Lesion in the early evolutionary stage with multiple violaceous lacunae and mild brown pigmentation. (B) Different lesion in the late regressive stage with central scar surrounded by whitish streaks and an eccentric focus of residual pigmentation.

The lesions presented with variable clinical and dermoscopic appearance depending on the stage. The most striking finding was the presence of reddish lilac pigmentation of nodular lesions with the presence of peripheral violaceous vessels resembling a solar eclipse (Figure 1). The majority of small papules showed multiple violaceous lacunae and clods over a light brown background; others presented a central scar-like area surrounded by whitish streaks (Figure 2).

**Figure 3:**
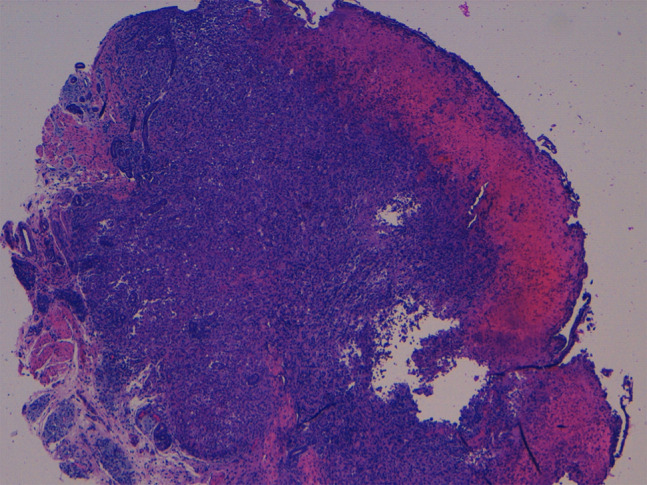
H&E-stained biopsy showing a dense dermal infiltrate of large histiocytes with pale cytoplasm and ectatic vessels in the dermis. Original magnification ×40.

**Figure 4:**
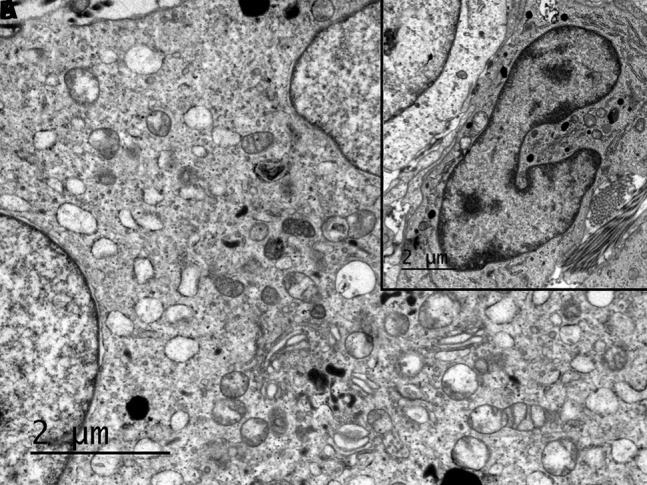
(A) Electron microscopy shows Birbeck and vermiform bodies. (B) Large histiocytes with reniform nuclei were also observed.

Histopathology confirmed the diagnosis of LCH, presenting diffuse proliferation of histiocytes in the reticular dermis surrounded by ectatic vessels (Figure 3). Immunohistochemical staining results for S100, CD1a, and langerin were positive. Electron microscopy showed large histiocytes with reniform nuclei and Birbeck and vermiform bodies (Figure 4).

## Conclusions

Dermoscopically characteristic lilac pigmentation and multiple burgundy round lacunae on the periphery were seen in our case. Especially when the lesions are in the fully developed stage, the dermoscopic image resembles a solar eclipse. There could be a parallel between this sign and the “setting sun” appearance described in early evolutionary and fully developed stages of juvenile xanthogranuloma (non-Langerhans cell histiocytosis) [2].
